# Engineering Human Mesenchymal Bodies in a Novel 3D-Printed Microchannel Bioreactor for Extracellular Vesicle Biogenesis

**DOI:** 10.3390/bioengineering9120795

**Published:** 2022-12-13

**Authors:** Richard Jeske, Xingchi Chen, Logan Mulderrig, Chang Liu, Wenhao Cheng, Olivia Z. Zeng, Changchun Zeng, Jingjiao Guan, Daniel Hallinan, Xuegang Yuan, Yan Li

**Affiliations:** 1 Department of Chemical and Biomedical Engineering, Florida A&M University (FAMU)-FSU College of Engineering, Florida State University, Tallahassee, FL 32310, USA; 2 High Performance Materials Institute, Florida State University, Tallahassee, FL 32310, USA; 3 Aero-Propulsion, Mechatronics and Energy Center, FAMU-FSU College of Engineering, Tallahassee, FL 32310, USA; 4 Department of Industrial and Manufacturing Engineering, FAMU-FSU College of Engineering, Florida State University, Tallahassee, FL 32310, USA; 5 Department of Pathology and Laboratory Medicine, David Geffen School of Medicine, University of California-Los Angeles (UCLA), Los Angeles, CA 90095, USA

**Keywords:** human mesenchymal stem cells, microchannel, wave motion, 3-D aggregates, extracellular vesicle biogenesis

## Abstract

Human Mesenchymal Stem Cells (hMSCs) and their derived products hold potential in tissue engineering and as therapeutics in a wide range of diseases. hMSCs possess the ability to aggregate into “spheroids”, which has been used as a preconditioning technique to enhance their therapeutic potential by upregulating stemness, immunomodulatory capacity, and anti-inflammatory and pro-angiogenic secretome. Few studies have investigated the impact on hMSC aggregate properties stemming from dynamic and static aggregation techniques. hMSCs’ main mechanistic mode of action occur through their secretome, including extracellular vesicles (EVs)/exosomes, which contain therapeutically relevant proteins and nucleic acids. In this study, a 3D printed microchannel bioreactor was developed to dynamically form hMSC spheroids and promote hMSC condensation. In particular, the manner in which dynamic microenvironment conditions alter hMSC properties and EV biogenesis in relation to static cultures was assessed. Dynamic aggregation was found to promote autophagy activity, alter metabolism toward glycolysis, and promote exosome/EV production. This study advances our knowledge on a commonly used preconditioning technique that could be beneficial in wound healing, tissue regeneration, and autoimmune disorders.

## 1. Introduction

Human mesenchymal stem cells (hMSCs) are coveted in clinical settings due to their immunomodulatory properties and secretion of trophic factors beneficial for the regeneration of damaged tissue [1]. Furthermore, hMSCs are relatively easy to isolate from source tissue, are seen as immune evasive, and are capable of self-renewal making them ideal as candidates for future allogenic “off the shelf” products [2,3]. As a result, demand for hMSCs and their derived products has grown exponentially over the past decade, highlighting the need for robust expansion processes capable of producing large quantities of therapeutically potent cells [4,5]. However, large-scale expansion and an inconsistent culture environment cause increased heterogeneity, rapid cellular senescence and gene alterations in hMSCs, leading to impaired functions and declined therapeutic efficacy [6,7,8]. To resolve this issue, a number of preconditioning strategies, such as hypoxia, oxidative reprogramming, and the use of pharmacological agents, have been proposed in order to preserve hMSC properties and restore the loss of therapeutic potential during culture expansion [9,10,11].

A promising preconditioning strategy for preserving hMSC cellular homeostasis is 3-dimentional (3D) aggregation. When cultured on a hydrophobic surface, hMSCs exhibit innate ability to simultaneously aggregate into 3D spheroids (mesenspheres). MSCs isolated from mesenspheres have been shown to be smaller in diameter, spherical in morphology, and have a reduction in cytoskeletal and extracellular matrix composition [12]. Compared to planar culture, mesenspheres display increased immunomodulatory, anti-inflammatory, and angiogenic properties while maintaining increased stemness and improved survival rates post-transplantation [12,13,14]. Current methods to generate hMSC aggregates or mesenspheres either lack the ability to control aggregate size or are not scalable beyond a laboratory setting. Furthermore, diffusion limitation and hMSC aggregate diameter play a prominent role in regulating cellular heterogeneity within aggregates. Kouroupis and Correa suggest that most spheroids can be divided into three zones [13]: an outermost layer, where MSCs are metabolically active and continue to proliferate; an intermediate zone consisting of MSCs in a quiescent state with relatively smaller nuclei than MSCs in the outermost shell of the spheroid; then an innermost layer consisting of a senescent/apoptotic core due to cells reaching the limit of nutrient/waste diffusion into and out of the core. However, there is debate in the literature over the diameter in which spheroids cross the threshold in the development of a necrotic core [13]. In fact, mesenspheres have been found to undergo metabolic reconfiguration, which results in altered oxygen metabolism negating the formation of a necrotic core [15,16]. Specifically, aggregate size has been linked to oxygen consumption rates, mitochondrial fragmentation, as well as glycolytic metabolism [15]. To date, the majority of hMSC aggregation studies have only compared traditional 2D cultures to 3D aggregation and have barely explored the dynamic vs. static aggregation process [17,18,19,20,21,22].

Recently, it has been demonstrated that hMSC therapeutic benefit is not through direct cell engraftment on damaged tissue sites but through their secretion of microRNAs, growth factors, cytokines, etc. [23]. Specifically, paracrine mediators such as extracellular vesicles (EVs), including a subset of small EVs known as exosomes, have garnered significant interest as a “cell-free” alternative in regenerative medicine that could bypass safety concerns associated with the injection of cell products such as vascular occlusions [24,25,26,27]. Exosomes are roughly 30–150 nm in diameter and contain microRNAs, mRNAs, proteins, lipids and metabolites etc. derived from the parent cells and can be taken up by recipient cells, transferring their therapeutic cargos and regulating cell–cell communications [28,29]. Beyond the cargo derived from the parent cell, EVs can also be engineered to carry specific cargos or drugs through transfections, sonication, electroporation, and other physical methods [30]. Their relative safety and ability to cross the blood–brain barrier make them ideal candidates as therapeutics in regenerative medicine and preclinical studies of exosomes for stroke, heart disease, and wound healing are already underway [31,32,33].

To elevate the 3D aggregation culture system capable of controlling hMSC aggregate properties for a consistent therapeutic outcome while simultaneously scaling to meet growing hMSC demands in clinical applications, this study developed a novel bioreactor system utilizing a wave motion bioreactor and 3D printing microchannel technology to facilitate size-controlled mesenspheres and corresponding exosome productions. The microchannels allow for direct control of cell numbers in the aggregates while helping to induce aggregation through cell–cell interactions. The dynamic wave motion created by miniaturized wave motion reactor further enhances cell–cell contact and assists hMSC aggregation [17,34]. The wave motion bioreactor has already been applied for the industrial scale-up of cell expansion or aggregation [35]. This study addresses changes in hMSC properties and EV secretion arising from static vs. dynamic aggregation and provides implications in the manufacturing of hMSCs and corresponding EVs for regenerative medicine to treat diseases such as heart disease and ischemic stroke.

## 2. Materials and Methods

### 2.1. hMSC 2D Culture

Frozen hMSCs from passage 0 to 2 were delivered from the Tulane Center for Gene Therapy. hMSCs isolated from the bone marrow of multiple de-identified healthy donors from age 19–49 years (Table S1) were collected from plastic adherence, with less than 2% expression of CD3, CD14, CD31, CD45 and CD117, greater than 95% expression of CD73, CD90, CD105 and CD147, while simultaneously possessing tri-lineage differentiation potential upon in vitro induction [36]. hMSCs (1 × 10^6^ cells/mL/vial) were cryopreserved in a culture media encompassing α-MEM, 2 mM L-glutamine, 30% fetal bovine serum (FBS) and 5% dimethyl sulfoxide (DMSO). hMSCs were thawed and seeded at 2500 cells/cm^2^ in a complete culture media containing α-MEM, 10% FBS (Atlanta Biologicals, Lawrenceville, GA, USA), and 1% penicillin/streptomycin (Life Technologies, Carlsbad, CA, USA) in a standard culture incubator at 37 °C at 5% CO_2_. The culture medium was changed every two days. Cells were grown to 80% confluence and harvested with a 0.25% trypsin/ethylenediaminetetraacetic acid solution (Invitrogen, Grand Island, NY, USA) at 37 °C for 5–7 min. Harvested cells were re-plated at a density of 2500 cells/cm^2^ and sub-cultured up to passage 4–6 for experiments.

### 2.2. 3-D Printing of the Microchannels

Designs for microchannels were developed in Solidworks software. Files were imported into Slic3r, and 3D-printed using fused deposition modeling of natural PLA filament with a DREMEL^®^ DigiLab 3D Printer 3D45 using a nozzle temperature of 200 °C and a bed temperature of 60 °C. Following printing, polydimethylsiloxane (PDMS) precursor and curing agent were poured into the mold and heated at 37 °C overnight. The following day, the mold and cast were separated. The channels formed in the PDMS mold were subsequently filled with an additional thin layer of PDMS precursor and curing agent and heated at 37 °C overnight in order to smooth out the channels and remove any imperfections. Channels were then sterilized overnight while being soaked in ethanol. The following day, the ethanol was removed and the channels were washed with sterile PBS. Channels were then coated overnight in a 5% bovine serum albumin solution to act as an antifoulant.

### 2.3. hMSC Aggregate Formation under Wave Motion

For dynamic aggregates, 200 µL of total media was added to the microchannels during aggregate formation. The seeding density was between 15,000 and 50,000 cells per channel. Upon addition of cells and media, microchannels were placed on a rocking base of a WAVE bioreactor (Xuri Cell Expansion System W5, GE Healthcare) in a standard humidified incubator (37 °C, 5% CO_2_) at a rocking angle of 8° and rocking speed of 20 rocks/minute for 72 h [17]. For static aggregates, 15,000 and 50,000 cells were seeded in each well in 200 µL of culture medium in an ultra-low attachment (ULA) 96-well plate with a round bottom (Corning, Corning, NY, USA) for 72 h. For the EV isolation experiments, aggregates were pooled together in EV depleted culture media (i.e., the growth medium containing EV-depleted FBS through ultracentrifugation [37]) and transferred to ULA 6-well plates into three wells each containing 2 mL of EV-depleted media. Dynamic aggregates were placed on the WAVE bioreactor at a rocking angle of 8° and rocking speed of 20 rocks/minute for 72 h.

### 2.4. DNA Assay for Cell Number Determination

Cell concentrations were determined with a Quant-iT™ PicoGreen kit (Invitrogen, Grand Island, NY, USA), following the methods reported previously [38,39,40]. Briefly, cells were harvested, lysed overnight using proteinase K (VWR, Radnor, PA, USA) at 50 °C, and stained with PicoGreen (Molecular Probes, Eugene, OR, USA). Fluorescence signals were read using a Fluoro Count (PerkinElmer, Boston, MA, USA) and the cellular DNA of samples was quantified using a standard curve.

### 2.5. Image Analysis of hMSC Aggregate Morphology

Following formation of the aggregates in microchannels and ULA plates, images of hMSC aggregates were taken on day 1 through 3 using a Nikon Eclipse Ti-U inverted microscope and attached DS-Qi1 monochrome digital camera. Images were analyzed using ImageJ software to calculate aggregate diameters. Specifically, the captured images were converted to binary images using ImageJ software (http://rsb.info.nih.gov/ij (accessed on 1 January 2022)) and analyzed with the “particle analysis tool”. Through particle analysis in ImageJ software, the Feret’s diameter of each aggregate in the images can be calculated, which provides the average size of the aggregates (*n* = 10).

### 2.6. Reverse Transcription-Polymerase Chain Reaction (RT-PCR)

Total RNA was isolated using TRIzol following the vendor’s instructions (Invitrogen). Reverse transcription was carried out using 2 μg of total RNA, anchored oligo-dT primers (Operon) and Superscript III (Invitrogen). Primers for specific target genes were designed using the software Oligo Explorer 1.2 (Genelink) (Table S2). *β-actin* was used for normalization as the housekeeping gene after comparison with *GAPDH*. RT-PCR reactions were performed using a SYBR green PCR master mix on an ABI7500 instrument (Applied Biosystems). The amplification reactions were performed and the quality and primer specificity were verified. Fold variations in gene expressions were quantified using the ΔΔCt method: 2^−Δ(CtTreatment − CtControl)^, in comparison to the static culture.

### 2.7. EV Isolation

For EV isolation experiments, culture media were replaced by EV-depleted media. on day 5 and collected on day 7. Conditioned media were sequentially spun (500 g for 5 min, 2000 g for 10 min, 10,000 g for 30 min) to remove cell debris, apoptotic body, large vesicles, etc. Polyethylene glycol (PEG)-6000 was added to the supernatant to a final end ratio of 8% PEG and 0.5 M NaCl and stored for 24 h at 4 °C as previously demonstrated [41] in order to enrich EVs. The solution was spun at 3000 g for one hour and the supernatant was discarded. The remaining pellet was suspended in 1 mL PBS and ultracentrifuged at 120,000 g for 70 min at 4 °C. The EV pellet was then suspended in 200 µL PBS and disrupted using a benchtop shaker at 1500 rpm for 5 min. EVs were diluted to 10^8^–10^9^ particles per mL in PBS for nanoparticle tracking analysis.

### 2.8. Nanoparticle Tracking Analysis (NTA)

NTA was performed on the isolated EV samples in triplicate to determine size distribution and particle concentration, on a Nanosight LM10-HS instrument (NTA 3.4 Build 3.4.003, Malvern Instruments, Malvern, UK) configured with a blue (488 nm) laser and sCMOS camera [41]. For each replicate, three videos of 60 s were acquired with camera shutter speed fixed at 30.00 ms. To ensure accurate and consistent detection of small particles, the camera level was set to 13, and the detection threshold was maintained at three. The laser chamber was cleaned thoroughly with particle-free water between each sample reading. The collected videos were analyzed using NTA3.0 software to obtain the mode and mean size distribution, as well as the concentration of particles per mL of solution. Compared to the mean size, the mode size is usually a more accurate representation because the vesicle aggregates may affect the mean size.

### 2.9. Western Blot Assay

EV samples were lysed in a radio-immunoprecipitation assay buffer (150 mM sodium chloride, 1.0% Trition X-100, 0.5% sodium deoxycholate, 0.1% sodium dodecyl sulfate, 50 mM Tris, pH 8) with addition of Halt protease inhibitor cocktail (Fisher Scientific, Hampton, NH, USA). The supernatant was collected, and protein lysate concentration was determined and normalized to the lowest sample concentration. Proteins were separated by 12% BIS-Tris-SDS gels and transferred onto a nitrocellulose membrane (Bio-rad, Hercules, CA, USA) for blocking with 5% nonfat dry milk (*w/v*) in Tris-buffered saline (10 mM Tris-HCl, pH 7.5, and 150 mM NaCl) with 0.1% Tween 20 (*v/v*) (TBST) and incubated overnight with primary antibody (Table S3) at 4 °C. Next, the membranes were washed and incubated with IR secondary antibodies (LI-COR, Lincoln, NE, USA) for three hours at room temperature. Membranes were washed three times in TBST and imaged using the LI-COR Odyssey (LI-COR) system.

### 2.10. Transmission Electron Microscopy (TEM)

Electron microscopy imaging was used to confirm the morphology and size of EVs. Briefly, EV isolates were resuspended in 30 μL of filtered PBS. For each sample preparation, intact EVs (15 µL) were dropped onto Parafilm. A carbon-coated 400 hex mesh copper grid (Electron Microscopy Sciences, EMS) was positioned using forceps with coating side down on top of each drop for one hour. Grids were rinsed three times with 30 µL filtered PBS before being fixed in 2% paraformaldehyde (PFA) for 10 min (EMS, EM Grade). The grids were then transferred on top of a 20 µL drop of 2.5% glutaraldehyde (EMS, EM Grade) and incubated for 10 min. Samples were stained for 10 min with 2% uranyl acetate (EMS grade). The samples were then embedded for 10 min with a mixture of 0.13% methyl cellulose and 0.4% uranyl acetate. The coated side of the grids were left to dry before imaging on the transmission electron microscope HT7800 (Hitachi, Japan).

### 2.11. Statistical Analysis

Data were analyzed in Graphpad Prism software. Data shown are represented as mean and standard deviations. Statistical analyses were performed with a Student’s *t*-test for the comparison of two groups (Figure 3) and significance was accepted at *p* ≤ 0.05. One-way ANOVA and a Tukey’s post hoc test were performed for multiple comparisons (Figures 4 and 5), and significance was accepted at *p* < 0.05. Experiments were performed at least three times in triplicate (*n* = 3), unless otherwise indicated.

## 3. Results

### 3.1. Microchannel Design

**Figure 1:**
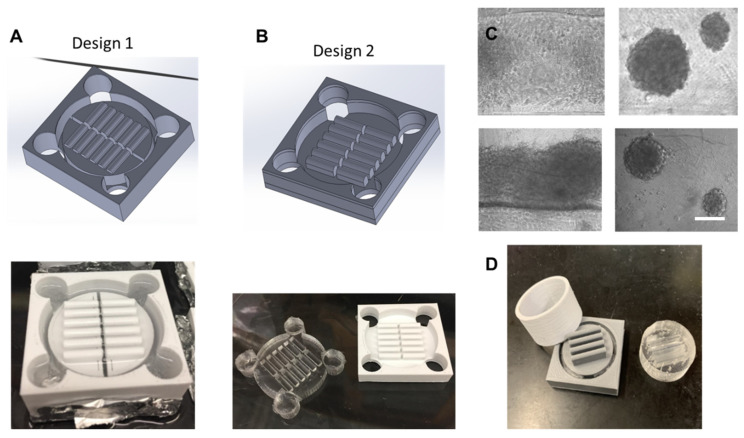
**3-D printed microchannel design.** Multiple design iterations of the microchannel reactor. (**A**) First design iteration in SOLIDWORKS and 3D print filled with PDMS. (**B**) Second design iteration in SOLIDWORKS and 3D print filled with PDMS. (**C**) hMSCs attaching to bottom of PDMS channel. Scale bar: 200 µm. (**D**) Final design iteration of 3D mold and PDMS cast.

The design process for the microchannels took multiple iterations in order to create a design capable of using polydimethylsiloxane (PDMS) as a surface substrate while being pragmatic for hMSC aggregation formation. Figure 1 shows multiple iterations of the design process. The first iteration shown in Figure 1A was unable to remove the PDMS mold from the cast as the adhesive forces were too strong. The second iteration shown in Figure 1B was designed to have lower walls allowing the PDMS cast to be pushed through the mold and removed. However, the channel design was not optimized and while aggregates did form, the majority of cells attached to the surface of the PDMS mold as shown in Figure 1C. The final design of the mold shown in Figure 1D shows a secondary cylinder that can be removed from the main piece. Upon removal from the main piece, the PDMS cast can then be pushed through the cylinder intact. This design allows for deeper microchannels that can hold enough media for fluid momentum to overcome liquid surface tension, facilitating aggregate formation.

**Figure 2:**
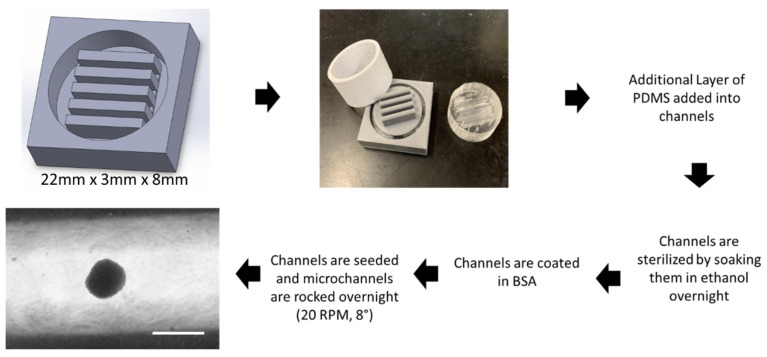
**3-D printed microchannel bioreactor and wave motion fluid dynamics.** Schematics detailing the preparation of hMSC aggregates. Initial draft of design is fabricated in SOLIDWORKS. Design is 3D-printed using DREMEL^®^ DigiLab 3D Printer 3D45. PDMS is added to the 3D mold forming the 6-well insert. An additional layer of PDMS is added to the microchannels smoothing out the surface. Channels are sterilized by soaking them in ethanol overnight under ultraviolet light. Channels are washed twice with PBS and then incubated with 5% bovine serum albumin (BSA) solution. Channels are seeded with hMSCs and rocked overnight at 20 RPM and 8° forming the aggregates. Scale bar: 200 µm.

Figure 2 outlines the processing technique to fabricate the PDMS cast and generate the hMSC aggregates. The first step is to design the 3D-print geometry in SOLIDWORKS. The final design utilizes flat microchannels 22 mm × 8 mm × 3 mm. The 3D mold is printed and filled with PDMS precursor reagent and allowed to crosslink overnight. An additional thin layer of PDMS is then added into each channel in order to smooth out the bottoms from any imperfections in the 3D print. Once the channels are completely formed, 70% ethanol is added to each and allowed to sit overnight under ultraviolet light for sterilization. Channels are washed with PBS, and then coated in a 5% bovine serum albumin solution as an antifoulant. A defined number of hMSCs were then seeded into each microchannel and cultured on the miniaturized wave reactor at 20 RPM and 8° overnight to form aggregates (the platform is shown in Figure S1).

### 3.2. MSC Aggregate Formation in 3-D Microchannel Bioreactors

**Figure 3:**
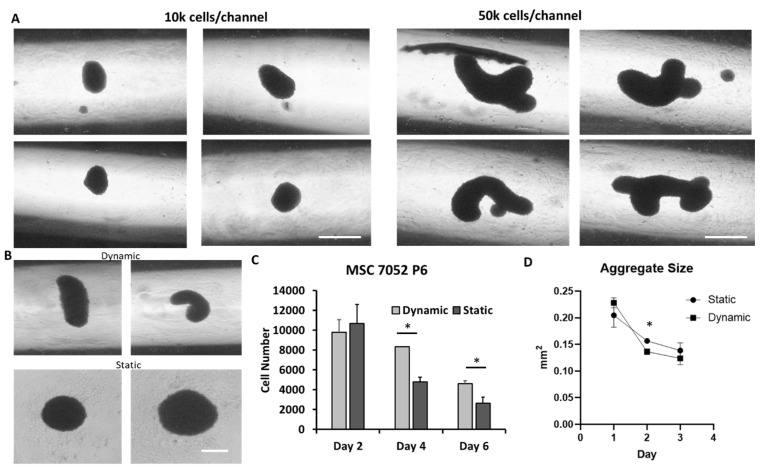
**HMSC aggregate morphology and characterizations.** (**A**) Aggregate formation at different seeding density. Scale bar: 200 µm. (**B**) Aggregate morphology for dynamic and static culture. Scale bar: 200 µm. (**C**) hMSC aggregate cell number change over time. (**D**) hMSC aggregate size change over time. hMSC aggregate size and cell number decrease throughout culture period. Dynamically cultured aggregates decrease size at a faster rate than their static counterparts. Cell number remains higher in dynamic aggregates through six days of culture compared to static culture. * indicates *p* < 0.05.

For better hMSC aggregate formation in the microchannel and in order to prevent the aggregate adhesion to the channel, the effect of surface coating was investigated (Figure S2). The round bottom and the no coating or using bovine serum albumin to block the cell adhesion was found to support hMSC aggregate formation. Figure 3 shows the aggregate size and shape through the three days of culture. At low seeding density (10 K cells/well), the hMSC aggregates were more spherical, while at high seeding density (50 K cells/well), the hMSC aggregates were more irregular and in a cylindrical morphology (Figure 3A). Compared to the static culture at the same seeding density, the dynamic culture had more irregular and cylindrical aggregates while the static culture had more spherical spheroids (Figure 3B). Interestingly, it was found that the cell number decreased faster in static culture compared to dynamic culture (Figure 3C), which may be due to a more significant diffusion limitation in static culture. In addition, the formation of 3-D aggregates significantly increased caspase 3/7 activity of hMSCs [42,43], which indicates potentially activated apoptosis. It was found that dynamically cultured aggregates decreased in diameter at a higher pace than statically cultured aggregates (Figure 3D), possibly due to hMSC condensation [44]. It was probably due to their initial morphology being elongated and becoming more spherical as the microchannels facilitate a more compact morphology.

### 3.3. MSC EV Production in Microchannel under Dynamic Culture

**Figure 4:**
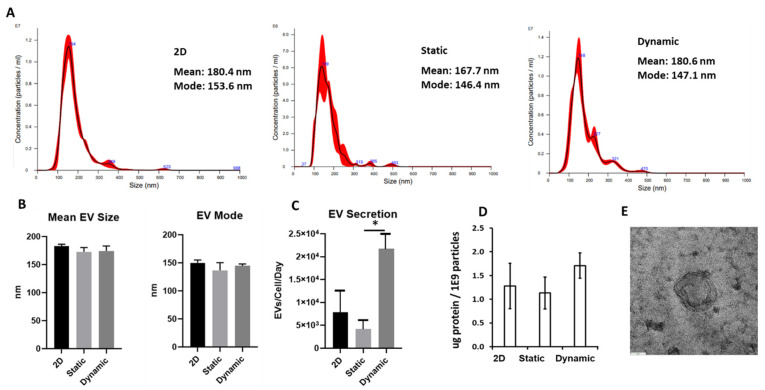
**Characterizations of the isolated EVs by nanoparticle tracking analysis (NTA) and protein assay.** Dynamic aggregation increases hMSC-EV secretion. (**A**) NTA analysis of EV size distributions. (**B**) Mean EV size; (**C**) Dynamic aggregation increases EV secretion roughly 5.2-fold over static aggregation and 2.7-fold over the 2D control. (**D**) Protein content normalized to EV number. (**E**) Transmission electron microscopy image for the isolated EVs of dynamic 3D culture. Scale bar: 60 nm. * indicates *p* < 0.05.

EVs were isolated from conditioned media of the aggregates and a 2D control. NTA revealed that the size distributions between the 2D and aggregate-secreted EVs had no statistically significant differences in mean and mode sizes. Mean EV diameters ranged between 165 nm and 185 nm while mode sizes ranged between 125 nm and 155 nm (Figure 4A,B). There was no statistical difference in average mean size and mode size among the different groups. Dynamically cultured aggregates had the highest EV secretion increasing roughly 5.2-fold compared to the static and 2.7-fold compared to the 2D group (Figure 4C). The protein content was also comparable for the three groups (Figure 4D) The exosomal markers Syntenin-1 and CD81 were expressed in the isolated EVs determined by western blot (Figure S3). TEM images showed the typical concave cup-shape morphology of exosomes, verifying the presence of exosomes in the isolated nanoparticles (Figure 4E).

### 3.4. Metabolism, Autophagy, and EV Biogenesis Marker Expression

**Figure 5:**
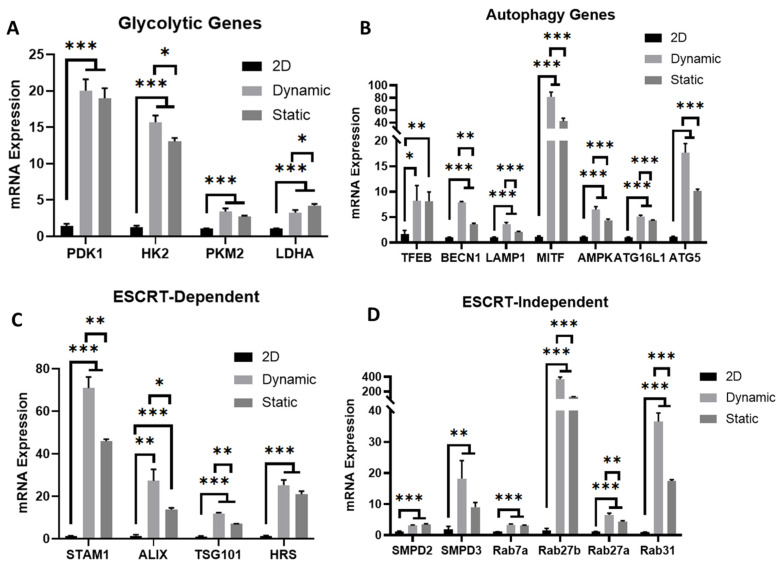
**Expression of metabolic markers and EV biogenesis markers.** mRNA expression of EV biogenesis markers in the cells were determined by RT-PCR. (**A**) Genes related to cellular metabolism; (**B**) Genes related to autophagy; (**C**) EV biogenesis markers-ESCRT dependent; (**D**) EV biogenesis markers-ESCRT independent. N = 3. * indicates *p* < 0.05; ** indicates *p* < 0.01, *** indicates *p* < 0.001.

To identify the potential mechanism in the promoted EV secretion in dynamic culture, genes related to cellular metabolism, autophagy, and EV biogenesis were determined by RT-PCR. Upon 3D aggregation, hMSCs exhibit metabolic plasticity to reconfigure their central energy metabolism towards glycolytic phenotype [19,34,38,43]. Moreover, glycolytic phenotype generally represents a primitive stage of stem cells in vivo [45]. In this study, hMSCs cultured in dynamic microchannels exert significant upregulation (by 4–20-fold) of glycolytic genes (*PDK1, HK2, PKM2, LDHA*) compared to 2D planer culture after three days of culture (Figure 5A). Compared to the static culture, a slight increase in *HK2* and *LDHA* was observed, indicating that the 3D cellular organization contributes more to the glycolysis than the dynamic microenvironment. With 3D aggregation in dynamic culture, hMSCs exhibited more active autophagy, characterized by the upregulation (5–80-fold) of a set of autophagy genes (*TFEB, BECN1, LAMP1, MITF, AMPK, ATG16L1, ATG5*) (Figure 5B). Interestingly, though cultured under a similar 3D environment, the dynamic culture under wave motion further enhanced the hMSCs’ autophagic activity, indicating that hydrodynamics can further impact hMSC homeostasis under a 3D culture.

Previous studies have demonstrated that non-adherent culture of hMSCs improves cell secretome, especially EV secretion [34,46]. The EV biogenesis can be dependent on the endosomal sorting complexes required for transport (ESCRT) pathways and can also be ESCRT-independent [47,48]. Under a microchannel culture, genes of both ESCRT-dependent (*STAM1, ALIX, TSG101, HRS*) (Figure 5C) and ESCRT-independent (*SMPD2, SMPD3, Rab7a, Rab27a, Rab27b, and Rab31*) pathways (Figure 5D) responsible for EV biogenesis were highly activated (by 10–70-fold and 5–40-fold, respectively) in hMSCs, especially for Rab27b (more than a 40-fold increase). Similarly, wave motion further upregulated EV biogenesis genes compared to the static culture, for all the four ESCRT-dependent genes as well as *Rab27a, Rab27b*, and *Rab31*. These results suggest that 3D culture with hydrodynamics alters hMSC cellular behaviors and thus enhances EV production.

## 4. Discussion

The development of a scalable bioreactor, capable of expanding hMSCs and the secreted EVs without loss of therapeutic potency, would fuel the currently unprecedented demand for cell/EV quantities that are bottlenecking hMSC-based clinical trials [49,50]. Furthermore, the scalability of this solution lowering production costs, coupled with the wide range of diseases hMSCs and the secreted EVs are effective in treating, could lead to “off the shelf” cell or cell-free therapy products providing unprecedented access to therapeutics to patients of lower socioeconomic status [51,52]. The hMSC-secreted EVs have been recognized as a promising therapeutic option for various tissue repair and drug delivery approaches [25]. However, low yield and scalable production of EVs remain big challenges for clinical applications.

3D printing for rapid prototyping has been in use in a number of engineering fields ranging from electronics to polymer processing [53,54,55]. In tissue engineering, 3D printing has seen further applications in the construction of patient-specific 3D scaffolds [56]. This study demonstrated an application utilizing the rapid prototyping of 3D prints in future hMSC and the secreted EV production. In the span of two weeks, a new PDMS cast could be designed, fabricated, and seeded with hMSCs for their preconditioning through aggregation. The optimal channel length was found to be 22 mm with a height of 8 mm. These dimensions allowed for the movement of 200 µL of media in the channel necessary for aggregate development. Optimal channel width was found to be 3 mm. When channel widths were less than 3 mm, capillary action prevented the movement of media in the channel necessary for aggregate formation. A width greater than 3 mm allowed for only three channels per 6-well insert.

Interestingly, microchannel aggregation initially generated more irregularly shaped or cylindrical-shaped aggregates than the static aggregation method. However, the dynamic movement in the microchannel led to a faster decrease in aggregate diameters (i.e., accelerated hMSC condensation) as the microchannel helped to shape the aggregates to be more spherical throughout the three days of culture. Compared to the spherical shape, irregularly shaped aggregates may have beneficial impacts upon the aggregate such as greater surface area to volume ratios allowing greater levels of nutrient and waste diffusion into the aggregate. Coupled with decreased mass transfer gradients associated with dynamic cultures, microchannel hMSC aggregates may have a more viable core free from necrosis leading to greater proliferative capacity and greater EV production [57]. Indeed, our results found the cell number to fall roughly 60% in the dynamic microchannel environment after six days while falling nearly 80% in static aggregation.

The hMSC aggregate formation was affected by cytoskeleton organization (Figure S4). Cytochalasin D (cytoD, an actin polymerization inhibitor), Y-27632 (ROCKi, a Rho-associated protein kinase (ROCK) signaling inhibitor), and nocodazole (Noco, a tubulin production inhibitor) were added into culture media, respectively. Compared to the control, the hMSC aggregates were smaller or looser for CytoD and ROCKi treatments. For Noco treatment, hMSCs cannot form compact spheroids after day 1. These observations indicate that actin-mediated contractility influences hMSC aggregation, compaction, and fusion [42,43]. It is suspected that the presence of microchannels and the use of wave motion accelerate the process of cytoskeleton organization.

Moreover, static aggregates generated similar amounts of EVs per cell to the 2D control while dynamically generated aggregates produced roughly 2.7-fold more EVs than the 2D control, when normalized to cell number on a per-day basis. The isolated EVs had an average diameter of 150 nm (mode size) or 180 nm (mean size). Increased EV generation from dynamic aggregation has been reported in literature previously [58], and Cha et. al. reported over a 100-fold increase in EV generation over their 2D control using dynamic aggregation. However, they reported increased static EV generation compared to the 2D control which was not observed in this study. To reveal the mechanism, our results indicate that EV biogenesis markers were upregulated in the dynamic culture of hMSC aggregates compared to 2D culture, consistent with our previous studies using microcarrier-based VerticalWheel bioreactors, microcarrier-based suspension bioreactors, and the wave motion aggregate culture in the absence of microchannels [34,40,59]. However, in the previous studies there were two parameter changes compared to the 2D control: (1) 3D cellular organization and (2) the dynamic culture environment (i.e., the presence of shear stress). This study separates these two effects by including the static 3-D aggregate culture. Compared to 2D culture, static 3-D culture showed an increased EV biogenesis, revealing the contribution of 3D architecture [46]. Compared to static 3-D culture, dynamic 3-D culture also increased the EV biogenesis, showing the contribution of the hydrodynamics of bioreactors [40,60].

The metabolic pathways were shifted toward glycolysis from oxidative phosphorylation in dynamic 3-D culture, which may be more attributed to the 3-D cellular organization than bioreactor hydrodynamics (Figure 5A). Moreover, hMSC aggregate condensation (accelerated by the dynamic microchannel bioreactor) has been linked to oxygen consumption rates, mitochondrial fragmentation, as well as glycolytic metabolism [61]. The autophagy genes were additionally upregulated in the dynamic aggregation culture, consistent with our previous observations in other dynamic culture systems [34,59]. It is suspected that there is a link between autophagic flux and EV production in the dynamic culture [62,63,64]. Autophagy activation can protect hMSC from apoptosis and trigger integrated stress response [19,64]. The EV secretion may also inhibit the autophagy activity as the feedback response [62].

This study demonstrates the feasibility of using a 3D printed microchannel bioreactor under wave motion to produce hMSC aggregates (or mesenspheres, mesenchymal bodies [65]) and the secreted EVs in a lab scale. The scalability of the microchannel bioreactor is yet to be demonstrated and associated with the 3D-printing capability and further optimization of the microchannel design. The effect of rocking speed and angle in the wave motion on the EV secretion is not well known either. In addition, the use of serum-free medium in the hMSC culture and the EV collection is preferred. This platform is more suitable for promoting hMSC aggregate condensation (Figure 3D) compared to the dynamic wave culture in the absence of a microchannel. To increase the number of hMSC aggregates, the dimensions of the microchannel require further optimization.

## 5. Conclusions

This study focused on aggregation as a preconditioning technique to enhance hMSC therapeutic properties. A 3D-printed microchannel bioreactor was developed to form hMSC spheroids to assess how dynamic microenvironment conditions alter hMSC properties and EV biogenesis. Dynamic aggregation was found to promote hMSC exosome/EV production compared to the static aggregate culture. In addition, the results separate the effect of 3D cellular organization and the dynamic culture environment on the EV biogenesis. Continuation of this study is a unique opportunity to assess the exact impact of hydrodynamics on EV secretion from hMSC aggregates as size, matrix substrate, and cell densities can be controlled exactly. Together, these results advance our understanding of bio-manufacturing of hMSCs as well as the secreted EVs and could have impacts in developing therapeutics for Alzheimer’s disease, ischemic stroke, and other neurological disorders.

## Data Availability

Available upon request.

## Supplementary Materials

The following supporting information can be downloaded at: https://www.mdpi.com/article/10.3390/bioengineering9120795/s1, Figure S1: Wave motion bioreactor for hMSC aggregate formation; Figure S2: Effect of coating on microchannel on aggregate formation; Figure S3: Western blot for exosomal markers for the isolated EVs; Figure S4: hMSC aggregation affected by cytoskeleton organization. Table S1: Donor information of bone marrow derived hMSCs; Table S2: Primer information for RT-qPCR analysis; Table S3: Antibody information [66].
