# Supplementary material for: Engineering Human Mesenchymal Bodies in a Novel 3D-Printed Microchannel Bioreactor for Extracellular Vesicle Biogenesis
Source: Bioengineering (Basel). 2022 Dec 13;9(12):795. doi: 10.3390/bioengineering9120795 (PMC9774207; doi:10.3390/bioengineering9120795)
Supplement: Supplementary file 1 [file bioengineering-09-00795-s001.zip › bioengineering-1967385-supplementary.pdf]

Engineering Human Mesenchymal Bodies in a Novel 3D-Printed Microchannel Bioreactor for Extracellular Vesicle Biogenesis

Richard Jeske [1], Xingchi Chen [1,2], Logan Mulderrig [1,3], Chang Liu [1], Wenhao Cheng [1], Olivia Z. Zeng [1], Changchun Zeng [2,4], Jingjiao Guan [1], Daniel Hallinan [1], Xuegang Yuan [1,5,*] and Yan Li [1,*]

[1] Department of Chemical and Biomedical Engineering, Florida A&M University (FAMU)-FSU College of Engineering, Florida State University, Tallahassee, FL, 32310, USA
[2] High Performance Materials Institute, Florida State University, Tallahassee, FL, 32310, USA
[3] Aero-Propulsion, Mechatronics and Energy Center, FAMU-FSU College of Engineering, Tallahassee, FL, 32310, USA
[4] Department of Industrial and Manufacturing Engineering, FAMU-FSU College of Engineering, Florida State University, Tallahassee, FL, 32310, USA
[5] Department of Pathology and Laboratory Medicine, David Geffen School of Medicine, University of California-Los Angeles (UCLA), Los Angeles, CA, 90095, USA
* Correspondence: yuanxg1989@g.ucla.edu (X.Y.); yli4@fsu.edu (Y.L.); Tel.: +850-410-6320 (Y.L.); Fax: +850-410-6150 (Y.L.)

Citation: Jeske, R.; Chen, X.; Mulderrig, L.; Liu, C.; Cheng, W.; Zeng, O.Z.; Zeng, C.; Guan, J.; Hallinan, D.; Yuan, X.; et al. Engineering Human Mesenchymal Bodies in a Novel 3D-Printed Microchannel Bioreactor for Extracellular Vesicle Biogenesis. *Bioengineering* **2022**, *9*, x. https://doi.org/10.3390/xxxxx

Academic Editor(s): Cornelia Kasper, Dominik Egger, Fedor Senatov, Michael Raghunath, Farhad Chariyev-Prinz

Received: 28 September 2022
Accepted: 9 December 2022
Published: date

Publisher's Note: MDPI stays neutral with regard to jurisdictional claims in published maps and institutional affiliations.

Copyright: © 2022 by the authors. Submitted for possible open access publication under the terms and conditions of the Creative Commons Attribution (CC BY) license (https://creativecommons.org/licenses/by/4.0/).

Supplementary

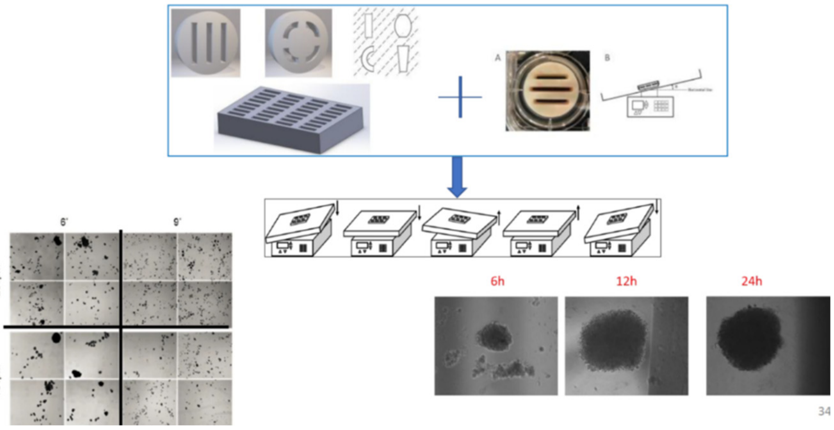

Figure S1. Wave motion bioreactor for hMSC aggregate formation. From reference: [1, 2].

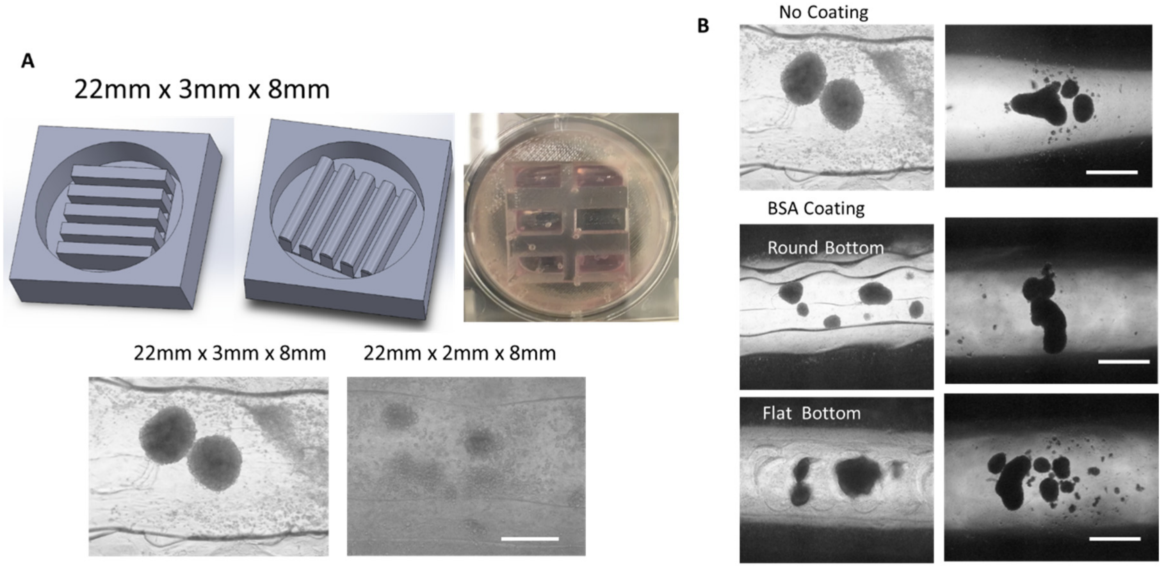

Figure S2. Effect of coating on microchannel on aggregate formation. (**A**) The microchannel dimension and the existence of aggregate adhesion in the channel; (**B**) The effect of bovine serum albumin (BSA) coating in the microchannel with round bottom and the flat bottom. Scale bar: 200 μm.

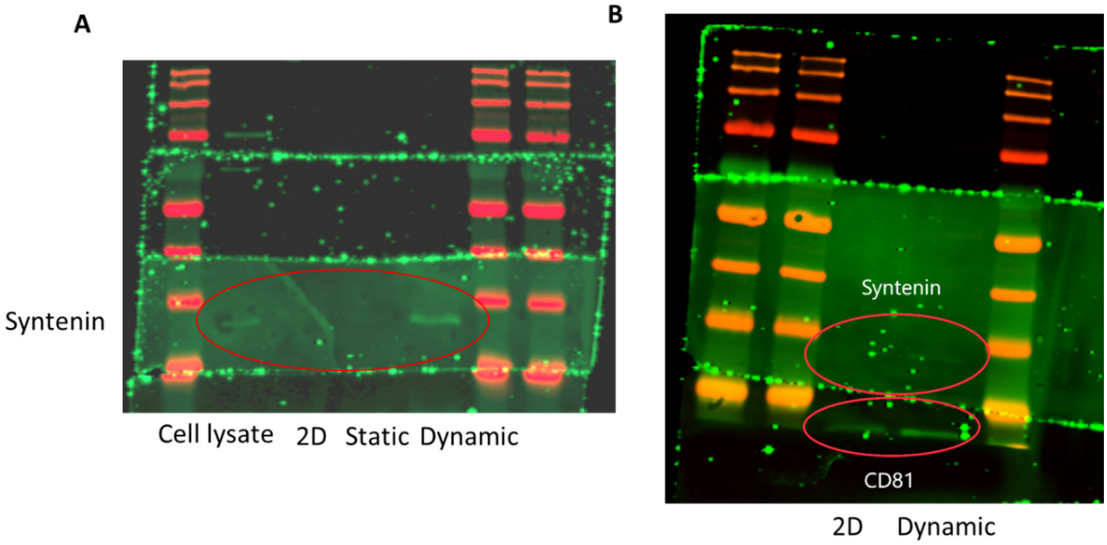

Figure S3. Western blot for exosomal markers for the isolated EVs. (**A**) and (**B**) are two different runs for different samples.

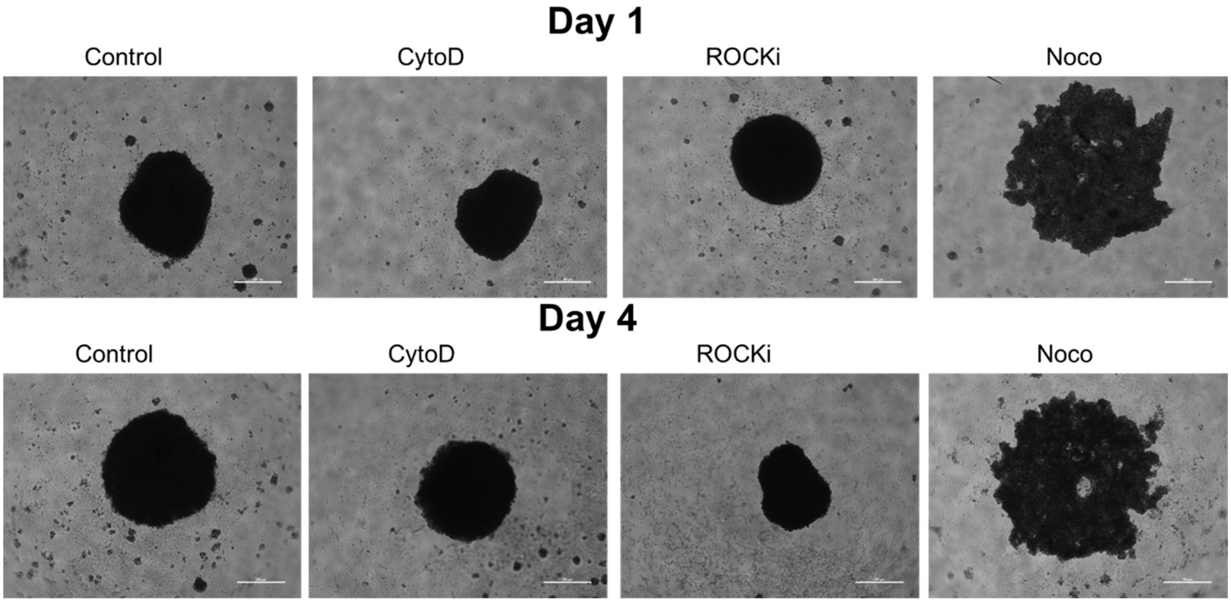

Figure S4. hMSC aggregation affected by cytoskeleton organization. Different inhibitors were added to the hMSCs (seeded at 5000 cells per well of low attachment 96-well plate) at day 0. Images were taken at day 1 and 4. Cytochalasin D (cytoD), Y- 27632 (ROCKi), and nocodazole (Noco) were added into culture media at final concentrations of 0.2 μM, 10 μM, and 1.0 μM, respectively. Scale bar: 100 μm.

Table S1. Donor information of bone marrow derived hMSCs.

ID	Years	BMI	Race	Sex	Note
7051R	33 yo	NA	caucasian	F	Tulane
7052R	20 yo	NA	caucasian	M	Tulane
7038	30 yo	NA	caucasian	F	Tulane

Table S2. Primer information for RT-qPCR analysis.

Primer Number	Gene Name	Primer Name in Database	Primer Sequence (5'-3')
	ACTB	Bactin F	GTACTCCGTGTGGATCGGCG
		Bactin R	AAGCATTTGCGGTGGACGATGG
	GAPDH	GAPDH-F	TCACTGCCACCCAGAAGACTG
		GAPDH-R	GGATGACCTTGCCCACAGC
1	PDK1	PDK1-F1	AAACAGGGGAGCTTTGTCTGG
		PDK1-R1	CTGCCCATTCACATCCCTCTA
2	HK2	HK2_F1	TGGTGTAGCTCCTCTGCTGCT
		HK2_R1	TGTGGGCACCCTTTAGTGAAC
3	PKM2	PKM2-F1	AAAAATGGATGCCCAGAGGAC
		PKM2-R1	GAGTCGGCTTCAATGGAACAA
4	LDHA	LDHA-F1	CCTTGAGCCAGGTGGATGTTT
		LDHA-R1	CACTGGATCCCAGGATGTGAC
5	TFEB	TFEB F1	CCTGGTGGAGATTCCCTGTCT
		TFEB R1	CAGGACCAGTTGCCTCAGATG
6	BECN1	BECN1 F1	ACTGTGTTGCTGCTCCATGCT
		BECN1 R1	AACGGCAGCTCCTTAGATTTGT
7	LAMP1	LAMP1 F1	TCACACGTAGGACGCATGAAG
		LAMP1 R1	GAAGCGCTCCAGACACTCATC
8	PRKAA1(AMPK)	PRKAA1 F	GTGTGTCAAGGTTGCAACAGAA
		PRKAA1 R	ATTTGCATTGCCTCCCTTACC
9	ATG5	ATG5 F	CCAAGCCTCTGGGATTTTACC
		ATG5 R	GCGTACTCAAATGGGTCAACA
10	ATG16LI-1	ATG16L1-F1	ATCTTTCCGTTTCAGGGGTTG

		ATG16L1-R1	GTTTCGCACACCCAATGAACT
11	SMPD2-1	SMPD2-F1	GCCTGGGAGACTTTCTGAACC
		SMPD2-R1	AAGTGGTGTGCAGCTGGGTAG
12	SMPD3-1	SMPD3-F1	TTAAGAGACTCCAGGGCTGCTC
		SMPD3-R1	CGGGGATTGTCAAAAACAGTC
13	SRSF5(hrs)-1	SRSF5-F1	CTTCTCGGATCGAGGCTTCTT
		SRSF5-R1	TCGAATCAACTGCGCTCATTA
14	TSG101	TSG101 F	CACCTGGTGGTCCATATCCTG
		TSG101 R	GATGGTGTCCTCGCTGATTGT
15	STAM1	STAM1-F1	CACTGGATTTTTGGGTTGCTC
		STAM1-R1	GTGGAAAACATTTTTCGCATGA
16	PDCD61P (ALIX)	PDCD61P F	TAAGTGCATCTGAGGGCCAAA
		PDCD61P R	GGGGCCTCCTTTCCTAGTTTC
	PDCD61PI4 (ALIXI4)	PDCD61Pi4 F	TTGGCTAATCAGGCTGCAGAT
		PDCD61Pi4 R	TCACATGCAAAGTAA-GCAAGTGTT
17	MITF-1	MITF-F1	GAATTGGTGATGGGTGATGGA
		MITF-R1	TGCATGGGAACTATGCAGTTG
18	RAB27A	RAB27A F	GCATGTTTCAGTTTTCAA-GAACCA
		RAB27A R	AAAGGTGGCTTTTGTGTGTGC
19	RAB27B	RAB27B F	TCCATGAAGCTGCTTGTCTCA
		RAB27B R	GTTGGGTCTCCACCCAGAAAT
20	RAB31	RAB31-F1	TCAAGGACTTTGGCATGTGGT
		RAB31-R1	TCACCATTCAACTTGCCATGA
21	RAB7A	RAB7A-F1	TGCTCCCTTCCTAGGATCTGC
		RAB7A-R1	CAGAAGAACTCAGCCCACACC

Table S3. Antibody information.

Primary Antibody	Origin/ Isotype	Supplier/Cat#	Dilution
CD81	Rabbit IgG	Cell Signaling Technologies, #56039	Western blot: 1:1000
Syntenin-1	mouse monoclonal IgG1	SANTA, CRUZ/sc-100336	Western blot: 1:1000
**Secondary Anti-body**	**Origin/ Isotype**	**Supplier/ Cat#**	**Dilution**
IRDye® 800CW	Goat anti-Rabbit IgG (H + L)	LI-COR/ 926-32211	Western blot: 1:5,000

References:

17. Tsai, A.C.; Liu, Y.; Yuan, X.; Chella, R.; Ma, T. Aggregation kinetics of human mesenchymal stem cells under wave motion. *Biotechnol. J.* **2017**, *12*, 1600448.
66. Yuan, X.; Tsai, A.C., Farrance, I.; Rowley, J.; Ma, T. Aggregation of Culture Expanded Human Mesenchymal Stem Cells in Microcarrier-based Bioreactor. *Biochem Eng J.* **2018**, *131*, 39–46.
